# Food supplements increase adult tarsus length, but not growth rate, in an island population of house sparrows (*Passer domesticus*)

**DOI:** 10.1186/1756-0500-4-431

**Published:** 2011-10-21

**Authors:** Ian R Cleasby, Terry Burke, Julia Schroeder, Shinichi Nakagawa

**Affiliations:** 1 Department of Animal and Plant Sciences, University of Sheffield, Sheffield, S10 2TN, UK; 2 Department of Zoology, University of Otago, Dunedin, PO Box 56, New Zealand

## Abstract

**Background:**

Variation in food supply during early development can influence growth rate and body size in many species. However, whilst the detrimental effects of food restriction have often been studied in natural populations, how young individuals respond to an artificial increase in food supply is rarely investigated. Here, we investigated both the short-term and long-term effects of providing house sparrow chicks with food supplements during a key period of growth and development and assessed whether providing food supplements had any persistent effect upon adult size (measured here as tarsus length).

**Results:**

Male nestlings tended to reach higher mass asymptotes than females. Furthermore, brood size was negatively associated with a chick's asymptotic mass. However, providing food supplements had no influence upon the growth rate or the asymptotic mass of chicks. Adults that received food supplements as chicks were larger, in terms of their tarsus length, than adults that did not receive extra food as chicks. In addition, the variation in tarsus length amongst adult males that were given food supplements as chicks was significantly less than the variance observed amongst males that did not receive food supplements.

**Conclusions:**

Our results demonstrate that the food supply chicks experience during a critical developmental period can have a permanent effect upon their adult phenotype. Furthermore, providing extra food to chicks resulted in sex-biased variance in a size-related trait amongst adults, which shows that the degree of sexual size dimorphism can be affected by the environment experienced during growth.

## Background

An individual's phenotype is a product of their genes and the environment in which those genes are expressed. Therefore, the environmental conditions experienced during development are important in determining the course of an individual's future phenotype and the course of their life-history [1,2]. For example, rearing individuals in poor environments can result in compromised phenotypic development [3]. On the other hand, individuals reared in a good environment can benefit from so-called 'silver spoon' effects; the positive, long-term effects of being reared in a good environment, which persist throughout the rest of their life [4,5].

One of the most important environmental causes of variation in growth and development is the food supply (in terms of both quantity and nutritional quality) received during [6,7]. Because wild animals often experience fluctuations in their food supply as they grow, a range of behavioural, morphological and physiological adaptations may have evolved to reduce the impact that variation in food supply has upon growth [8]. How individuals should alter their growth strategy as their food supply changes has been widely studied and a number of potential responses have been identified [8]. In some species food restriction leads to lower rates of growth and, consequently, to delayed maturation [9]. In such circumstances, individuals may increase the length of their growth period in order to reach a normal adult size [10,11]. Alternatively, if the food supply improves following a period of food limitation, individuals may undergo a period of 'catch-up' or compensatory growth during which growth rate is accelerated relative to their age or state. The existence of compensatory growth suggests that growth rate is optimised rather than maximized and can vary depending upon the prevailing circumstances [1]. Rapid catch-up growth may incur costs such as a decline in cognitive performance [12], an increased risk of certain health problems [13], and potentially a reduction in lifespan [14], which may explain why individuals do not necessarily grow at the maximum rate possible all the time.

Birds have proved a successful model system for the study of growth and development [15]. Indeed, the relationship between food supply and avian growth and development is well-established [8]. However, while many studies have focussed upon how food limitation can alter growth rates and final body size in natural populations [7,16], how chicks respond to an increase in their food supply has rarely been studied despite calls for such an approach [8]; but see [17]. In the pied flycatcher (*Ficedula hypoleuca*) providing chicks with mealworms during the course of one breeding season did not alter the growth rate of chicks or their final size [18]. Styrysky et al [19] found that supplemental food increased nestling mass in the house wren (*Troglodytes aedon*), but only in early-season broods. In the starling (*Sturnus vulgaris*), supplemental food had a positive effect on nestling tarsus length [20]. In the magpie (*Pica pica*) food supplementation appeared to the influence the heritability of tarsus length in some cases but had no effect in others [21,22]. Other experiments have shown that providing chicks with specific nutrients such as extra calcium, or extra vitamin E, can increase their growth rate and size at fledging [23,24]). However, few studies have been able to measure the effects of food supplementation on adult size.

How individuals respond to extra food may have important evolutionary consequences. For example, because the heritability of growth and body size tend to be lower under poor conditions [25] genotype by environment interactions could shape the evolution of particular traits [26]. Moreover, if the sexes differ in their sensitivity to the food supply received during growth this could influence patterns of sexual dimorphism in animal populations [27].

In the present study we investigated both the short-term and long-term effects of providing nestling house sparrows with supplemental food. To assess the short-term impact of food supplements on nestling growth we calculated the growth rate and final, asymptotic body mass of house sparrow (*Passer domesticus*) chicks using logistic growth curves [28] and compared the performance of chicks that received food supplement with those that did not. To investigate the long-term effects of food supplementation we examined whether adults that were fed as chicks were larger (in terms of tarsus length and mass) than those individuals that did not receive extra food as chicks. The house sparrow is an ideal species to use for such analyses because food supply has already been identified as a factor that limits nestling growth and survival in this species [29,30].

## Results

### Growth analysis

**Figure 1:**
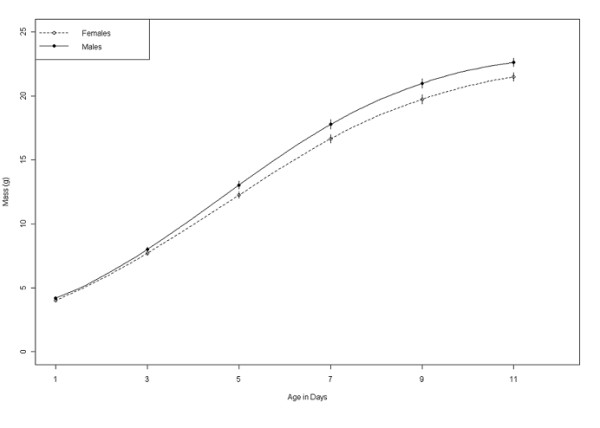
**The average mass (g) ± SE of males and females at each age based upon the raw data, *n = *138 females, 123 males**.

The results of our non-linear regression show that the growth parameters estimated from Lundy are comparable with previous studies on the house sparrow (Table 1). Out of a total of 19 candidate models tested only three were included in our confidence set, together the combined AIC weight of these three models was 0.96. Our experimental treatment had very little effect upon the asymptotic weight (*A*) that individuals reached or their growth rate (*K*) (Table 2). Instead, we found that the asymptote of males was significantly higher than that of females (Figure 1; Table 2), but mass at 1 day old did not differ between males and females (*t*-test: mean mass of males = 4.17 (g) ± *SD *1.23, *n *= 123; mean mass of females = 4.00 (g) ± *SD *1.10, *n *= 138; *t *= 1.19, *p *= 0.24). However, despite reaching a greater asymptote, males grew at the same rates as females (Table 2). Individuals reached smaller asymptotes when they were reared in large broods (Table 2), but brood size had no influence upon an individual's growth rate. The rate of growth increased as the season progressed; individuals that hatched in the Mid- and Late-season had a higher growth rate than individuals hatched early in the season. Growth rate also varied by year, and in 2007 the growth rate was lower than it was in both 2008 and 2009 (Table 2), however, the summed AIC weight of the Year term in our models was weak (0.08). There was a strong correlation between an individual's mass at 11 days old and their estimated asymptote (*r *= 0.93, 95% CI = 0.91-0.95, *df *= 258), which suggested that our model accurately represented chick growth.

**Table 1:** House sparrow growth parameters in different studies estimated using non-linear least squares.

Reference:		*A*	*K*
Dunn (1975)^§ ^		25.0	0.393
Schifferli (1980)^§ ^	female	23.8	0.425
	male	24.8	0.443
This study	female (138)	23.0 ± 0.48	0.417 ± 0.02
	Male (123)	24.1 ± 0.47	0.434 ± 0.02

*A *= asymptotic mass (g) ± SE, *K *= growth rate ± SE, sample size is given in parentheses. § Data taken from Starck and Ricklefs [15]; sample sizes and standard errors were not reported.

**Table 2:** Model averaged growth curve results from a non-linear mixed effects model of growth.

Model Parameter	Coefficient	95% Confidence Interval	Summed Weight
*A *(Intercept)	27.91	25.56-30.25	N/A
*A *~ Sex (Male)	1.03	0.31-1.75	0.96
*A *~ Brood Size	-0.64	-1.26--0.03	0.99
*A *~ Treatment (Fed)	0.04	-1.09-1.16	0.35
*A *~ Mid-Season	-1.07	-2.89-0.75	0.99
*A *~ Late-Season	-0.44	-1.76-0.88	0.99
*A *~ 2008	1.23	-1.02-3.48	0.08
A ~ 2009	-1.15	-2.85-0.56	0.08
*B *(Intercept)	12.20	11.47-12.92	NA
*K *(Intercept)	0.365	0.190-0.541	NA
*K *~ Sex (Male)	-0.001	-0.018-0.016	0.96
*K *~ Brood Size	-0.009	-0.023-0.006	0.99
*K *~ Treatment (Fed)	0.011	-0.017-0.039	0.35
*K *~ Mid-Season	0.090	0.009-0.172	0.99
*K *~ Late-Season	0.100	0.012-0.190	0.99
*K *~ 2008	0.100	0.061-0.139	0.08
*K *~ 2009	0.110	0.071-0.149	0.08

The intercept represents a female reared in the control group. *n = *261 chicks from 103 broods. Summed weight represents the summed AIC weight of all models that include a particular parameter within the total set of candidate models. Where *A *is the asymptotic size, *K *the rate constant of the equation and *B *is the constant of integration

Tarsus length at 11 days old did not differ between control and supplemented chicks (Table 3). In addition, there was no evidence that the tarsus length of male and female chicks differed. Tarsus length was positively associated with hatch date and with a chick's mass at one day old. Finally, the variance in tarsus length did not differ between males in the control group and males in the treatment group (variance ratio = 1.14, 95% CI = 0.68-1.89).

**Table 3:** Model averaged estimates of the factors affecting tarsus length at 11 days old.

Parameter	Coefficient	95% Confidence Interval	Summed AIC Weight
Intercept	17.60	17.40-17.60	N/A
Brood Size	-0.06	-0.21-0.10	0.53
Sex (Male)	0.11	-0.10-0.32	0.51
Day 1 Mass	0.42	0.21-0.56	1.00
Treatment (Fed)	0.05	-0.32-0.42	0.42
Hatch Date	0.012	0.005-0.020	1.00

### Adult measurements

**Figure 2:**
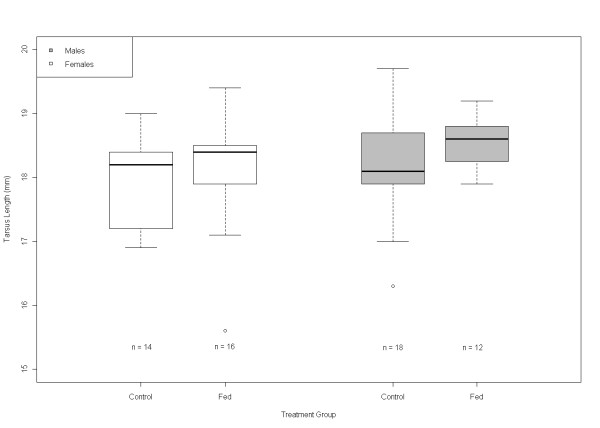
**Adult tarsus length measurements for males and females that successfully recruited into the population in the different treatment groups**. The bold line in the middle of the boxes is the median, the boxes show the interquartile range (IQR), which goes from the first quartile (the 25^th ^percentile) to the third quartile (the 75^th ^percentile). The whiskers go from 1.5 times the first quartile to 1.5 times the third quartile.

Providing chicks with supplemental food had a positive effect upon adult tarsus length; individuals that were provided with supplemental food as chicks tended to have a longer tarsus as adults than chicks that did not receive supplemental food (Table 4). In addition, there was less variation in adult tarsus length amongst males that were given supplemental food than amongst males that were not (Table 5; Figure 2), but not in females. In contrast, food supplements had no effect upon the mass of birds once they reached adulthood (Table 6).

**Table 4:** Model averaged estimates of the factors affecting adult tarsus length.

Parameter	Coefficient	95% Confidence Interval	Summed AIC Weight
Intercept	18.19	17.85-18.54	N/A
Brood Size	-0.04	-0.13-0.04	0.29
Sex (Male)	0.11	-0.05-0.26	0.38
Treatment (Fed)	0.14	0.07-0.21	0.44
Hatch Date	0.03	-0.04-0.10	0.22

**Table 5:** Variance ratio in adult tarsus length between the different sexes and treatment groups.

Variance Test	Variance Ratio	95% CI of Ratio	*p*
Control Males vs. Fed Males	3.51	1.10-9.55	0.03
Control Females vs. Fed Females	0.61	0.21-1.83	0.37
Control Males vs. Control Females	1.16	0.39-3.06	0.79
Fed Males vs. Fed Females	4.99	1.51-14.65	0.01

Sample sizes given in Figure 2. Raw *p*-values from *F*-tests and confidence intervals are reported here. When comparing the ratio of two variances a value of one means that they are equal.

**Table 6:** Model averaged estimates of the factors affecting adult mass.

Parameter	Coefficient	95% Confidence Interval	Summed AIC Weight
Intercept	26.61	25.94-27.28	N/A
Brood Size	0.16	-0.35-0.67	0.19
Sex (Male)	0.53	-0.25-1.31	0.66
Treatment (Fed)	0.10	-0.70-0.90	0.32
Hatch Date	0.02	0.01-0.04	0.02
Season	-0.31	-1.17-0.55	0.18
Time of Day	0.006	0.004-0.008	1.00

## Discussion

We found that food supplementation influenced the variance observed in adult tarsus length in the house sparrow, but had no effect upon asymptotic size or growth rate. While food availability has often been identified as a major source of variation in growth, this has typically been demonstrated by restricting an individual's access to food. Using such an approach, Richner et al. [31] showed that carrion crow chicks (*Corvus corone*) experiencing food restriction grew more slowly and were smaller as adults. In other species, the rate of maturation may be reduced, resulting in a delay in the timing of feather eruption and a lengthening of the nestling period [9]. The mechanism by which food restriction alters growth seems relatively straight-forward, a lack of food equals a lack of the basic materials required for growth and development. In contrast, how chicks respond to the provision of extra food or nutrients is less clear. We were not able to alter the growth rate or asymptote of house sparrow chicks by providing them with supplemental food, nor was there an effect upon tarsus length at 11 days old. Similarly, Verhulst [18] reported that providing collared flycatcher nestlings with mealworms had no effect upon their mass at fledging or their growth rate. However, other studies have shown that dietary supplements can influence growth rate and final size [23,24], although these studies focused on a specific nutrient rather than a more general supplement as we did here.

One reason why our supplement may have had no effect upon a chick's growth rate is that food is not a limiting factor in this population. However, this is unlikely because our food supplementation increased chick survival in smaller broods [32]. An alternative explanation is that the amount of the supplement given to chicks was not large enough to have an impact upon a chick's growth rate. In a series of experiments, Lepczyk and Karasov [33] subjected house sparrow chicks to a period of 48 hours of food restriction and then supplied them with food *ad libitum*. Although food restricted chicks consumed 15% more food than control nestlings (which had experienced no food restriction) when fed *ad libitum*, they were unable to convert this extra food into a faster growth rate. In the song sparrow, control chicks that were fed *ad libitum *grew faster than chicks limited to a food intake of 60% of the controls [11]. In general, it seems that in order to influence growth, a large amount of additional food would be required in most studies. Moreover, even with a plentiful food supply there may be costs associated with an increased growth rate [1]. For example, individuals growing at faster rates may be more susceptible to starvation [34], or suffer from increased oxidative damage [35]. Indeed, in certain cases nestlings might actually be predicted to reduce their growth rate when possible [36]. However, nestlings could benefit from extra food via avenues other than increased growth rate. For example, the extra food could provide nestlings with essential nutrients that could then be devoted to key physiological functions such as immunocompetence or antioxidant defence. In the future it would be interesting to investigate how providing extra food alters investment in other key life-history traits beyond growth rate and body size.

Male house sparrows reached a higher asymptote than females, which concurs with the results of previous studies on the house sparrow (Table 1). However, the extent to which species exhibit sexual dimorphism may depend on the influence of the environment upon growth. For example, in the snow goose (*Chen caerulescens*) the extent of sexual dimorphism is reduced when individuals are reared in poor environments [37]. Such a process may also be occurring in the Lundy house sparrow population, during the years 2003 to 2006 we found no evidence of any sexual dimorphism in body mass, or tarsus length, between male and female fledglings [38], but in the present study (2007-2009) we did.

The other factor influencing an individual's asymptote was brood size, and we found that chicks from larger broods reached smaller asymptotes than chicks from smaller broods. The negative effect of brood size upon fledging mass has been found before. In particular, clutch size manipulation experiments often show that chicks reared in enlarged broods are smaller than those reared in control or reduced broods [39]. Our study shows that even natural variation in brood size will influence an individual's mass at fledgling. In the house sparrow the provisioning rate per chick drops as brood size increases [40]. Therefore, chicks from large broods may be smaller because the greater competition for food means that most individuals receive less food than they would if they were reared alone. Given that body mass is often highly heritable in passerines [25], and that fledgling mass can influence future fertility [41], the fact that brood size can influence mass demonstrates its importance as a source of variation in growth [42].

The only factors we found that influenced growth rate, were the time in the season that a chick hatched, and the year in which they were reared. Brood size had no effect upon growth rate, nor did an individual's sex. Hatch date has been identified as an important determinant of growth rate in previous studies [38]. The house sparrow is capable of breeding throughout the summer, but the quality of the food supply that chicks receive varies throughout the season depending on which insects are available [30], and this may influence chick growth rates. On Lundy, the survival of chicks born early in the season is slightly lower than that of chicks hatched later in the season [38]. Furthermore, of the early-hatched chicks that do survive to fledge many appear small and under-weight at 11 days old (IR Cleasby, pers obs.). Therefore, chicks born early in the season may have a lower rate of growth because they receive less food.

Although males reach a higher asymptote than females, growth rate does not differ between the sexes. In fact, even in species with much more extreme sexual dimorphism than the house sparrow, the growth rate of males and females often does not differ [6]. Because growth rate varied between the years to a larger extent than it did between treatment groups, or between the sexes it may have been difficult to detect an effect of both these variables in the face of large between-year variation in the environment. We do not know why growth rate was faster in 2008 and 2009 than it was in 2007, but presumably this was due to differences in crucial ecological factors such as the weather or the abundance of insects in each year. The summed AIC weight for the year term in the model is relatively low however, which suggests that other variables may have been more important.

The environmental conditions experienced during growth can have a strong effect upon body size in birds [43]. Here, we found that males fed supplemental food exhibited less variation in tarsus length than control males, which suggests that food may influence skeletal development. We also found some evidence that food supplementation had a small, positive effect on adult tarsus length. A similar effect has also been documented in the magpie where De Neve et al. [21] found that the heritability of tarsus length was higher amongst food-supplemented chicks than control chicks. In contrast, we found little evidence that food supplementation influenced the mass of birds once they reached adulthood. One explanation for this could simply be that the food supply affects body structures in different ways [7]. If so, then the extra food we provided may have been utilised for skeletal growth rather than growth in general body mass. Alternatively, it may simply be difficult to detect the effect of supplemental food on adult body size simply because it fluctuates so much. Furthermore, because body mass is a composite measure of the mass of all the structures that make up an individual it does not allow us to discern if particular parts of the body were influenced by extra food.

Differences in average tarsus length and the variation in tarsus length between control and experimental chicks were not present at 11 days old. However, environmentally induced variation in tarsus length may be greater in fledglings than adults [44], and could mask the effect of the treatment on fledglings. For example, when we restricted our models of 11 day old tarsus to only include those individuals that survived to adulthood the effect of supplemental treatment on tarsus length was still apparent (see additional file 1). Because the difference in the variance of tarsus lengths between fed and control males were present at 11 days old, the results seen in adults were probably due to the influence of food supply during rearing rather than being caused by selection acting differently on control and treatment individuals post-fledging. Tarsus length is an important trait in the house sparrow as it may be under positive directional selection due to its positive association with male mating success [45]. The results suggest that food supply has the ability to alter an individual's phenotypic reaction norm [46] and potentially even their life-history strategy as a result.

The decrease in the variance of tarsus length amongst adult males suggests that nutrition during growth can alter the environmental component of a trait. Providing supplemental food seems unlikely to alter the additive genetic component of a trait, but we cannot rule out the possibility of epistatic interactions. Any change in the variation of a trait may have important consequences because interactions between environmental and additive genetic variance can influence evolutionary response [47]. For example, the narrow-sense heritability of size-related traits can be reduced in poor conditions because in poor conditions the environment may be responsible for more of the variation in a trait than the additive genetic variance [48]. Thus, changes in food supply may influence a population's ability to respond to selection pressures, or at the very least, our ability to reliably detect responses to selection.

A reduction in the variance of a trait such as tarsus length could be seen as an example of canalization. In its broadest sense, canalization refers to the process whereby the variability of a trait is reduced by a developmental mechanism ([49]; see [50] for other definitions). Typically, most studies of canalization monitor the development of individuals in response to environmental disturbances [51]. Our experiment differs from studies of canalization in that we sought to improve the environment an individual experienced during growth. The reduction in variation of tarsus length in males that were given extra food suggests that the development of this structure is not well-buffered against variation in food supply in males. In contrast, no such effect was observed in females. Why the variance in male tarsus length should be affected by our treatment, but not female tarsus length, is unclear. In the great tit, persistent environmental effects appear more prevalent in males than females [52], which suggests that males may be more sensitive to the environment in which they are reared (reviewed in [53]). The differential effects of the environment on the phenotype of males and female offspring are important because they may cause parents to adjust their investment in offspring on the basis of an offspring's sex [54].

## Conclusions

We have shown that the food supply experienced during growth influences the distribution of a size-related trait in adults. Crucially, the results suggest that the nutrition available to chicks as they grow has the capacity to shape evolutionary responses. In particular, we have demonstrated experimental evidence that extra food can act as a 'silver spoon' through its effect on skeletal size. As a result, measuring phenotypes may not give us an accurate impression of evolutionary response to selection unless we consider the influence of changing environmental conditions, such as food supply.

## Methods

### General procedures

The study was conducted over three years, from 2007 to 2009, on an house sparrow population on Lundy Island, England (51°10'N, 4°40'W). The population is based around a small village and farm, which are situated in the south-east corner of the island and cover an area of approximately 1 km^2^. During these three years, the Lundy house sparrow colony was small, fluctuating between 15-35 breeding pairs per year. Since 2000, this population has been subject to continuous, systematic monitoring, with almost all breeding birds and fledglings individually marked with unique colour band combinations and a metal ring supplied by the British Trust for Ornithology (BTO). Adult birds are caught throughout the whole breeding season (April-August), and also for one week during the winter (December-February). In addition, most breeding attempts occur in nestboxes that have been specially erected by us in and around various farm buildings. The house sparrow population on Lundy is relatively isolated. Lundy is 19 km away from the UK mainland with the sea representing a barrier to dispersal. House sparrows themselves are a sedentary species and the average dispersal is approximately 1.5-2 km. We have no records of any house sparrows ringed on Lundy subsequently being re-captured on the mainland and the rate of immigration to the island as been estimated as less than one bird per year [40,55].

### Experimental procedures

During the breeding season we monitored all breeding attempts that took place within nestboxes by regularly visiting them and monitoring their status. We recorded when eggs were laid in a particular nest and, after the 12^th ^day of incubation, we visited a nest once a day, every day, until the eggs hatched. We recorded the date on which the first egg in the brood hatched and then converted this to a numerical measure of hatch date using the 1^st ^of April to represent day 1 at the start of the breeding season. Breeding attempts that did not occur in nestboxes were not included in the experiment because the age of the chicks was unknown. At hatching chicks were defined as being 0 days old, we took our first morphological measurements (mass in grams and tarsus length in millimetres) of chicks when they were 1 day old, the day after they had hatched. Barkowska et al. [56] recommended using mass data from 6 out of the first 13 days of the nestling period to accurately characterize nestling growth in the house sparrow. Therefore, for our growth curve analyses we measured the mass of chicks every other day from day 1 through until day 11. Between days 1 and 11 we marked each chick in a brood distinctly by regularly trimming one of their claws every time we measured them, which allowed us to recognise different individuals before they were ringed. When chicks reached 11 days old, they were fitted with a metal BTO ring and a unique combination of colour rings for individual identification. Chicks were sexed using the primers P2 and P8 [57]. We also recorded whether or not a particular chick successfully survived over the nestling period (from 1 days old-11 days old).

When a brood hatched, we assigned it to a particular treatment group, all chicks within a brood either received supplemental food, or they did not. Broods were randomly allocated to a particular treatment group. However, because house sparrows typically raise more than one brood per year, we also tried to alternate which treatment an adult pair received between different broods. In most cases we successfully managed to alternate the treatment that an adult pair received broods. The only cases in which pairs received the same treatment two broods in a row occurred when we did not know the identity of the pair before beginning the treatment. In total, 7 pairs out of a total of 58 experienced the same treatment for consecutive broods.

Experimental broods were fed a suspension of nestling growth formula (Kay-Tee Exact Hand Feeding Baby Bird Formula, KayTee Product); this formula has been used before to feed house sparrow nestlings in the field [58]. Chicks received their first meal when they were two days old, and were fed every second day afterwards until they reached 11 days old. Food was mixed according to the specifications given by the manufacturers (formula diluted 3:1 in hot water until day 4 and 2:1 from day 5 onwards) and chicks received the supplemental food through a modified syringe with a hollow, rigid plastic tube ca. 6 mm attached at the bottom. For each brood, we fed chicks one at a time, giving each chick time to drain its crop, and then repeated this process feeding each chick once more. In total, chicks received 2 ml of this mixture (0.4-0.6 g of dry formula) over these two rounds of feeding. Based upon information from the manufacturer, the feeding supplement we used provided chicks with 16.4 kilojoules of energy per gram. Using the estimated daily energy budget (DEB) of a 1-11 day old house sparrow from Blem [59], which is 36.0-101.7 kJ, we estimated that our supplements provided chicks with approximately an extra 10% of their DEB every other day. All broods were fed and measured before midday and morphological measurements were taken before we began feeding chicks.

Control broods were also measured every other day starting from day 1, but they received no supplemental food. However, to control for the handling time involved in hand-feeding experimental chicks, we sham-fed control chicks. To do this, we held an empty syringe feeding tube to the mouth of each of the chicks in a brood for 30 seconds and then repeated this procedure after the chicks had been left for one minute. The feeding protocol described was devised by Mock et al. [58] and where more details can be found.

There was only one confirmed case of polygyny (a male caring for two broods simultaneously) observed over the three years of the study (IR. Cleasby pers. obs.). Removing these two broods from our analyses did not alter our results. Therefore, we included information from both polygynously-reared broods within our analyses. In total, we included 428 chicks from 125 broods in this experiment, 188 chicks from 54 broods received supplemental food and 240 chicks from 71 broods did not receive any food supplements. However, because we require six data points per chick in order to construct a growth curve only individuals that successfully fledged were included within our statistical analyses of growth. Overall, we were able to construct growth curves for 261 chicks (138 females and 123 males) from 103 broods, 127 chicks from 50 broods received extra food and 134 chicks from 53 broods did not. Our research followed the guidelines set out in the ASAB/ABS Guidelines for the Use of Animals in Research [60] and our experiments were approved by the University of Sheffield's Ethics Review Panel.

### Statistical analysis

All statistical tests described were carried out in the R environment, version 2.10.0 (R Development Core Team 2009) [61]. We began by testing the fit of our growth data to each of the growth equations described by Ricklefs [28]; these growth equations are commonly used to study avian growth and have been used to model the growth of house sparrow nestlings before [15,62]. The logistic growth equation was identified as the best fit to our growth data on the basis that it had the lowest Akaike information criterion (AIC) score. The logistic growth equation is as follows:

(1) $$S_{t}=A∕1+B*e\left(-K*\left(T\right)\right)$$

Where *S_t_*is the size of an individual at time *t*, *A *is the asymptotic size, *K *the rate constant of the equation, *B *is the constant of integration, that translates individuals to a common time-scale, and *T *represents time or age of an individual. The asymptote is the largest size that an individual reaches during growth. The growth rate *K *is the gradient of the logistic curve at its steepest point. Note that this equation is a slight variation of the equation provided by Ricklefs [28], but has been used previously in the literature [63]. Both versions of the logistic growth equation give exactly the same estimates for *A *and *K*, however, we preferred to use this modified version of the equation presented here because it resulted in quicker convergence of our statistical models. Logistic growth curves were fitted to our data using non-linear least squares, running separate models for males and females, using the nlme package [64]. This gave us mean population estimates of *A *and *K *for males and females that were comparable with previous studies of house sparrow growth (see Table 1).

To estimate growth curves a non-linear mixed effects model was used to account for repeated measures of individuals nested within broods. As predictors of the growth parameters *A *and *K *we included an individual's sex, experimental treatment (fed or control), brood size as measured on day 1, hatch date and the year of the study. Year was treated as a three-level factor. Furthermore, although hatch date can be coded as an integer, we experienced problems with model convergence when it was treated in this way. Instead, we converted hatch date into a 3-level factor (Early-season, Mid-season and Late-season) that denoted at what point in the season a brood hatched. These levels were determined by taking the difference between the latest hatch date observed and the earliest observed in a particular year, and then dividing this value by three in order to create three categories of an equal size. We included two-way interactions between experimental treatment and brood size, treatment and sex and treatment and year, on the basis of preliminary analysis and plots of our data following the protocols described in Zuur et al. [65]. *B *was also included in our model, but we did not include predictors for this term because we were only interested in estimating *A *and *K*. Initially our random effects structure was chick identity nested within brood identity nested within pair identity. However, models with this random effects structure failed to converge. We then compared models in which the random effects structure was chick identity nested within pair identity to those in which chick identity was nested within brood identity. There was no clear difference in the results obtained from either model, but the model in which chick identity was nested within brood identity had a much lower AIC score. Therefore, we included individual identity nested within brood identity as random effects in our models.

Initial residual plots of our model showed strong evidence of heteroscedasticity, and we found that the variance in chick mass was greater in older age classes. To model this, we used a power variance function with age set as a variance covariate, this improved model fit as estimated with AIC and via inspection of plots of the normalized residuals. Because of the nature of our data, 6 measurements per individual over time, we also identified evidence of significant auto-correlation. To model this, we included an AR-1 auto-correlation structure, which models the residual at time t (ε_t_) as a function of the residual at time t-1 (ρε_t-1_), along with a noise term (ηt) as follows:

(2) $$\varepsilon_{\text{t}}{=}_{\rho }\varepsilon_{\text{t}-1}+\eta_{\text{t}}$$

In this equation ρ represents the correlation between residuals one unit apart in time and must be estimated from the data, in our study ρ was 0.46. The AR-1 correlation structure means that the correlation between residuals decreases the further away they are in time. As well as the AR-1 correlation structure, we tested a number of auto-regressive moving average (ARMA) models following advice given in Zuur et al. [66], and we chose the AR-1 structure because it resulted in the model with the lowest AIC value.

Because mass growth only represents one particular type of growth we also assessed the effect that supplemental feeding had on a chick's tarsus length at fledging. To do this we used a linear mixed-effects model with a chick's tarsus length (as measured at 11 days old) as the response variable. The fixed effects included in the model were whether an individual received supplemental food or not, an individual's sex, the date an individual hatched, the size of the brood an individual was reared in as measured at day 1 and the individual's mass at one-day-old. As random effects we included brood identity nested within year.

We used linear mixed-effects model to measure the effect that food supplementation had upon adult phenotype. In one model adult tarsus length was used as the response variable. The fixed effects included in the model were: whether an individual received supplemental food or not, an individual's sex, the date an individual hatched and the size of the brood an individual was reared in as measured on day 1. As a random effect, we included the identity of the brood in which the individual was born nested within the year they were born. Initially, our model showed evidence of heteroscedasticity and graphical inspection of our data showed that the variance observed in adult tarsus length was influenced by both an individual's sex (Figure 2), and whether they received food supplements as a chick (Table 2). To solve this problem we used an identity variance covariate structure, which allowed us to set a different variance for each level of the sex by treatment interaction. This improved model fit, as based on AIC score and inspection of normalized residual plots.

We also ran another model in which the response variable was adult mass. The fixed effects included in the model were: whether an individual received supplemental food or not, an individual's sex, the date an individual hatched and the size of the brood an individual was reared in as measured on day 1 and the season (winter or summer) in which the measurements were taken. Because the mass of birds tends to fluctuate throughout the day we also included a fixed effect for time of day in the model. The time of day was coded such that 12 noon on a particular day was recorded as time 0, the time individuals were measured was then record as the number of minutes before or after 12 noon individuals were measured. For example, if an individual was measured at 11:55 am they would be scored as -5 minutes before 12 noon, whereas an individual caught at 12:05 pm would be scored as +5 minutes. We chose to centre our time-of-day variable at 12 noon so that the intercept in our model would have an easy to understand interpretation. As a random effect, we included the identity of the brood in which the individual was born nested within the year they were born.

We used a model-averaging approach based on AIC scores and using a natural-averaging technique [67] in order to identify the relative importance of variables in our models, and to generate weighted and less biased coefficient estimates [68]. We limited ourselves to testing up to a maximum of 20 candidate models and then reduced this to a confidence set of models, upon which we based our model averaging. Models were only included in the confidence set if their AIC weight was at least 10% of the highest AIC weight (e.g. if the best model had a weight of 0.6 then a model with a weight of 0.06 would be included in the confidence set). A list of the all the candidate models used can be found as supporting information in additional file 1.

## Competing interests

The authors declare that they have no competing interests.

## Authors' contributions

IRC and SN shared in designing the experiment and the statistical analyses. IRC collected the data in the field. IRC wrote the paper, SN, JS and TAB provided discussion and comments on earlier versions of the manuscript. TAB provided the funding. All authors read and approved the final manuscript.

## Supplementary Material

Additional file 1 **Details of all the candidate model sets used in the model averaging process and additional data analyses**. Click here for file
