# Supplementary material for: Food supplements increase adult tarsus length, but not growth rate, in an island population of house sparrows (Passer domesticus)
Source: BMC Res Notes. 2011 Oct 21;4:431. doi: 10.1186/1756-0500-4-431 (PMC3225329; doi:10.1186/1756-0500-4-431)
Supplement: Additional file 1 — Details of all the candidate model sets used in the model averaging process and additional data analyses. [file 1756-0500-4-431-S1.DOC]

**Additional File 1**

**Table A1 -** Candidate models used for growth curve analysis. Only the top three models were included within our confidence set. Key: BS = Brood Size, HD = Hatch Date, TR = Treatment, YR = Year

| Model ID Model Covariates ∆ AIC Model Weight |
| --- |
| (1) Sex, BS, HD 0 0.64  (2) Sex, BS, HD, TR 2 0.23  (3) Sex, BS, HD, TR, YR 4 0.08  (4) BS, HD, TR 6 0.03  (5) Sex, HD, TR 8 0.01  (6) Sex, BS 23 <0.001  (7) Sex 25 <0.001  (8) Sex, TR, BS 26 <0.001  (9) BS 27 <0.001  (10) BS, TR 30 <0.001  (11) Null Model 30 <0.001  (12) Sex, BS, Sex×BS 31 <0.001  (13) Sex, TR, Sex×TR 32 <0.001  (14) TR 33 <0.001  (15) Sex, BS, YR 33 <0.001  (16) Sex, TR, BS, YR 33 <0.001  (17) Sex, TR, BS, YR, TR×BS 33 <0.001  (18) Sex, TR, BS, YR, Sex×TR 91 <0.001  (19) Sex, TR, BS, YR, Sex×BS 92 <0.001 |
|  |
|  |

**Table A2 -** **A list of the candidate models used to model adult tarsus length. The top seven models were included within the confidence set. Key: RM = Relative Mass, BS = Brood Size, HD = Hatch Date, TR = Treatment.**

| Model ID Model Covariates ∆ AIC Model Weight |
| --- |
| (1) HD, BS, RM, Sex, TR 0 0.25  (2) HD, RM 0.32 0.22  (3) HD, BS, RM 1.16 0.14  (4) HD, RM, Sex 1.38 0.13  (5) HD, RM, BS, Sex 2.07 0.09  (6) HD, RM, TR 2.38 0.08  (7) HD, BS, RM, TR 3.24 0.05  (8) HD, RM, Sex, TR 3.47 0.04  (9) RM, Sex, TR 12.27 < 0.01 |

**Table A3. A list of the candidate models used to model adult tarsus length. The top** seven models were included within the confidence set. Key: RM = Relative Mass, BS = Brood Size, HD = Hatch Date, TR = Treatment.

| Model ID Model Covariates ∆ AIC Model Weight |
| --- |
| (1) TR 0 0.19  (2) Sex 0.02 0.18  (3) BS 0.45 0.15  (4) HD 0.60 0.14  (5) TR, Sex 1.17 0.11  (6) TR, HD 1.83 0.08  (7) BS, Sex 1.83 0.08  (8) BS, TR 2.34 0.06  (9) BS, HD, TR, Sex 7.62 < 0.01 |

**Table A4. A list of the candidate models used to model adult tarsus length. The top seven models were included within the confidence set. Key: BS = Brood Size, HD = Hatch Date, TR = Treatment, ToD = Time of Day, Seas = Season (Winter or Summer)**.

| Model ID Model Covariates ∆ AIC Model Weight |
| --- |
| (1) ToD 0 0.30  (2) ToD, Sex 0.81 0.20  (3) ToD, Seas 2.00 0.11  (4) ToD, TR 2.43 0.09  (5) ToD, Sex, Seas 2.80 0.07  (6) ToD, BS 3.17 0.06  (7) ToD, Sex, TR 3.29 0.06  (8) ToD, BS, Sex 3.89 0.04  (9) ToD, Seas, TR 4.56 0.03  (10) ToD, BS, TR 5.56 0.02  (11) ToD, HD, TR 7.77 0.01  (12) ToD, BS, HD 8.95 <0.01  (13) ToD, BS, HD 14.84 <0.01 |

**Table A5 - Variance ratio in 11 day old tarsus length between the different sexes and treatment groups. Here, the data has been restricted to include only those individuals that survived to adulthood. Sample sizes given in Figure** 2.

| Variance Test Variance Ratio 95% CI of Ratio *p* |
| --- |
| Control Males vs. Fed Males 3.36 1.03 – 9.66 0.04  Control Females vs. Fed Females 0.65 0.24 – 2.14 0.51  Control Males vs. Control Females 1.62 0.52 – 4.68 0.40  Fed Males vs. Fed Females 0.33 0.11 – 1.08 0.07 |

### Table A6 - Model averaged estimates of the factors influencing 11 day old tarsus length of those individuals that subsequently survived to adulthood.

| Parameter Coefficient 95% Confidence Interval Summed AIC Weight |
| --- |
| Intercept 17.94 17.40 – 18.47 N/A  Brood Size 0.003 -0.007 – 0.013 0.02  Sex (Male) 0.001 -0.01 – 0.01 0.02  Treatment (Fed) 0.15 0.05 – 0.25 0.40  Hatch Date 0.43 0.21 – 0.65 0.98 |

###

**Figure S1**. 11 day old tarsus length measurements for males and females that successfully recruited into the population. Sample sizes as reported in Figure 5.1 above.
